# Implementing laboratory automation for next-generation sequencing: benefits and challenges for library preparation

**DOI:** 10.3389/fpubh.2023.1195581

**Published:** 2023-07-13

**Authors:** Jillian N. Socea, Victoria N. Stone, Xiaorong Qian, Paula L. Gibbs, Kara J. Levinson

**Affiliations:** Division of Laboratory Services, Tennessee Department of Health, Nashville, TN, United States

**Keywords:** next-generation sequencing, automation, validation, verification, public health

## Abstract

In the wake of COVID-19, the importance of next-generation sequencing (NGS) for diagnostic testing and surveillance-based screening has never been more evident. Considering this, continued investment is critical to ensure more public health laboratories can adopt these advanced molecular technologies. However, many facilities may face potential barriers such as limited staff available to routinely prepare, test, and analyze samples, lack of expertise or experience in sequencing, difficulties in assay standardization, and an inability to handle throughput within expected turnaround times. Workflow automation provides an opportunity to overcome many of these challenges. By identifying these types of sustainable solutions, laboratories can begin to utilize more advanced molecular-based approaches for routine testing. Nevertheless, the introduction of automation, while valuable, does not come without its own challenges. This perspective article aims to highlight the benefits and difficulties of implementing laboratory automation used for sequencing. We discuss strategies for implementation, including things to consider when selecting instrumentation, how to approach validations, staff training, and troubleshooting.

## 1. Introduction

The introduction of accessible next-generation sequencing (NGS) technology has changed the landscape of clinical and public health microbiology. It offers the possibility of improving diagnostics, surveillance, and public health response. Sequencing can now be used to routinely support outbreak investigations, thus helping laboratories detect disease clusters sooner and with more clarity (1–3). By replacing standard microbiology methods with culture-independent applications for pathogen detection, NGS has the potential to guide more targeted patient care (4). Therefore, it is not surprising over the last 3 years there has been a major push to invest in genomic sequencing (5). NGS data has been essential during the COVID-19 pandemic. When combined with epidemiology, it offers a means to investigate transmission patterns as the virus continues to spread across the globe. Now, more than ever, clinical, and public health facilities are working with limited staff. New hires may lack the knowledge or experience to understand sequencing assays. Thus, at first glance, implementing NGS technologies may seem too complicated and time-consuming for laboratories to onboard or to even try to increase sequencing capacity. Workflow automation provides an opportunity to overcome some of these barriers. General laboratory automation has been used in many different types of laboratories for years (6), and has only exploded more recently, resulting in a multi-billion dollar market (7). As demand for next-generation sequencing increases, it only makes sense to consider how automation could potentially be used to support this type of testing. Here we discuss several aspects of automation for preparing sequencing libraries. We highlight the key benefits as well as some of the challenges of using automated liquid handlers. We also discuss how we approached validating one of these systems.

## 2. The benefits of NGS automation

Preparing specimens for next-generation sequencing is a time-consuming process, involving multiple steps, starting from sample extraction. The process of preparing the sequencing libraries is a critical part in obtaining high-quality results. It involves several time-sensitive steps, pipetting small volumes, as well as washing and re-washing samples. The entire process can take hours of a bench scientist’s time and one mistake can result in the loss of an entire day’s worth of work. Several manufacturers have designed automated liquid handlers specifically for this complicated process. Automated instruments of various sizes and capabilities have been created and can be programmed to perform an entire library prep protocol as a single streamlined process or in separated individual steps. While automation is not a magical solution to fix every problem, it does offer several benefits worth noting and may allow laboratories to overcome some of the hurdles involved with implementing NGS (8).

### 2.1. Improved quality

The most obvious reason for automation is enhanced sample quality, often with greater consistency than most laboratory scientists can reproduce manually. For NGS, a lot of library prep protocols use magnetic beads and repeated wash steps for purification and fragment size selection. Manual preparation requires that scientists are skilled and fully trained, otherwise samples may be lost, contaminated, or of suboptimal quality, all which affects downstream analyses. Automated platforms are designed for these precise pipetting steps, producing consistent high-quality libraries in less time than it takes using manual preparation. In our experience, we have observed quality improvements by a few measures including more uniform nucleic acid fragment lengths and less need for repeat testing of samples. Ultimately, a decrease in failed runs saves time, reagents, and supplies.

### 2.2. User friendly interface

Although the backend algorithms to automate a sequencing library preparation protocol can be complicated, many platforms come with a computer pre-programmed with user-friendly control software. Scientists do not need a lot of experience with NGS or a deep understanding of the scientific process to setup or run these liquid handlers. Established protocols often use simple images to display exactly where consumables should be placed, provide visual cues to indicate what step of the process is occurring, and the instrument can perform calculations to determine reagent volumes needed for the number of samples being run. Therefore, net training time is reduced, and scientists should not need specialized programming expertise to troubleshoot basic issues.

### 2.3. Increased flexibility

Automated instruments often allow laboratories to scale-up or scale-down as needed. There are instruments that offer variable levels of throughput while maintaining quick turnaround times. A lab can process 4–384 samples per run, depending on their system and needed output. Another added benefit is that some platforms offer modular workflow options with safe stopping points that enable labs to adjust as needed. Instead of an end-to-end process, labs can opt to only use the instrument for certain steps like library clean-up. Those that need more than the standardized library prep protocol offered through commercial vendors, manufacturers like Agilent and Beckman Coulter have graphical or simplified software interfaces that removes some of the complexity of creating customized protocols. They also offer training courses on method programming through their software. Hamilton and Beckman Coulter also have decks that can be reconfigured for new workflows. However, this may not be the case for all platforms. Some platforms have locked-in protocols that require the manufacturer to establish new workflows.

### 2.4. Timesaving

Automated platforms for library prep can perform more than just liquid transfer and mixing. Instruments can be customized to include on-deck thermocyclers, shakers, and heat blocks for a fully automated system, reducing the need for any manual interference. If prepared manually, the Illumina DNA Prep protocol takes approximately 3 h to generate a sequencing ready library. While the overall run time is similar for an automated workflow, the hands-on time is far reduced (approximately 30 min to set up instrument plus 2.5 h automated run time versus 3+ hours for the manual protocol per 8 samples processed). An added benefit is that only one scientist is needed to setup an instrument, regardless of the number of samples being run. Once the samples are loaded and the program is started, that scientist is free to walk-away and focus on other tasks.

## 3. The challenges with NGS automation

While automated workflows have many benefits, as mentioned above, there are some significant challenges to consider before deciding to implement such systems.

### 3.1. System cost, design, and setup

Automated instruments are often quite expensive to purchase (quotes we have received range between $45K–300K spanning a low throughput platform and two different high throughput instruments), and careful consideration should be given to determine whether one may be realistic or necessary for the current and future workload. Such systems have become increasingly complex, often with many add-on options like on-deck thermocyclers, bulk pipettor attachments, and robotic arms to suit different laboratory capacity needs and to perform various assay protocols. It is worthwhile to have a firm understanding of the fundamental components of a given procedure and/or product before settling on the system design that will be optimal for use. Depending on the starting sample material, reagent kit type, and sequencing platform to be used, there may be a limitation as to what instruments are compatible and available to choose from. We estimate it costs about $40 per sample, so there is likely little room to save in actual cost, but the relief in hands-on technician time may be worth it. Additionally, as automation becomes more widespread, costs may come down for consumables and for new systems in the future.

### 3.2. Troubleshooting and training

Initial on-site training is likely to be provided by a representative from the manufacturer to allow for familiarization with the installed instrument and to provide an overview of the basic workflow that it performs. However, hands-on experimentation will likely be required to gain experience and a better understanding of the system’s nuances. Clean water runs and test runs will help assess what steps and actions may present run errors or other issues that could impact the downstream quality of testing samples. Modifications to the software program running the workflow may be necessary to ensure that steps are performed accurately and seamlessly with minimal error and stoppage within the user’s laboratory. Although it’s worth noting that modifications may be limited by the manufacturer. In our experience, there is very little prospect of access to off-site vendor training to enable customization of software or protocol workflows. As with any new instrument, testing personnel will need to be trained. It may prove fruitful to train more senior staff (upper management or section supervisors) as “super users” to protect against loss of expertise to employee turnover or in times of limited availability of competent staff. We recommend maintaining a minimum of two “super users” at all times. These “super users” should be proficient in troubleshooting more difficult errors that may require remote assistance from the manufacturer, as well as the ability to realign deck positions (“deck teaching”), among other skills.

### 3.3. Routine performance and maintenance

One of the perks of using automated workflows is the concept of being able to “walk-away” without interruption to testing. The true experience though may be more complicated than that. Following manufacturer’s instructions for daily, weekly, and more long-term maintenance programs is crucially important to keep the instrument running smoothly. Routine maintenance includes channel calibration, both spacing, aspiration and dispensing, as well as surface cleaning to remove dust or other contaminates. This will likely be automatically prompted for by the instrument, but if not, a regular schedule (weekly) for such activities should be implemented. Annual preventative maintenance is also often provided by the manufacturer under special contracts (additional $15K–30K/year) to limit likelihood of bigger problems accumulating. These preventative maintenance appointments likely require scheduling with the on-site representative, which means there may be a waiting period before service is performed. The same is often true for any other service calls that may be needed when the instrument has an error or issue that the user is not able to resolve on their own. In our experience, direct communication with field engineers and applications specialists is common, which reduces instrument downtime and removes the need for tiered response through the general customer service line. Although our setup does not allow remote access, others may be able to design their system to enable this feature to limit the need for on-site visits when fixing minor errors and problems. Maintaining competency on a manual preparation method is recommended to ensure workflow is not halted if instrumentation requires repair or service.

### 3.4. Quality control within system limitations

As with all assays, quality controls (QC) must be continuously monitored to ensure the implemented instrument and protocol provides consistent, reliable, and accurate results. Within sequencing, there are often many QC “checkpoints” to confirm that each sample’s integrity is maintained from one step to another; these are often at key points in the procedure (e.g., after DNA extraction, after library preparation, after sequencing, etc.). Within automated systems, and depending on the library preparation kit used, there may be limitations in the ability to measure the sample quality at these predetermined points. In some cases, it may be necessary to modify appropriate timepoints for such system checks, and to be creative with when and how quality can be measured. For example, certain extraction/cell lysis methods may end with a beaded product, therefore, traditional quantification methods may not be practical after such steps. If situations like this arise, it becomes critical to establish quality thresholds at the next earliest available opportunity to limit time, sample, and reagent waste. In the instance that the extracted specimen cannot be measured due to the presence of beads, we find the QC checkpoint of quantifying DNA upon completion of the library preparation to be critical in determining whether each sample meets the quality needed for sequencing. This means that there may be reagent and sample waste if one does not meet the threshold for sequencing, and the sample will have to be completely re-extracted. This can be an annoyance in our experience, but it has not happened frequently or more often than in other methods.

## 4. One system does not fit all

There are a variety of automated platforms currently on the market geared toward next-generation sequencing library preparation (Table 1). Before committing, laboratories should assess their budget, facilities, and sequencing workflow to help identify what would work best for them to meet their sequencing goals. While a clinical lab may prioritize high-volume testing, a research facility may require a system with a flexible workflow. For this summary, we will focus on three main areas: system compatibility, system capability, and system capacity.

**Table 1:** Fully automated library preparation platforms.

Manufacturer and platforms	
Mid- to High-Throughput (up to 384 samples)	Agilent Bravo
	Beckman Coulter Biomek i-Series
	Eppendorf ep*Motion* ^®^
	Hamilton Microlab STAR^™^ Microlab VANTAGE^™^
	PerkinElmer Sciclone G3^®^ Fontus^™^ Zephyr^®^
	SPT Labtech Firefly^®^
	Tecan Fluent^®^ DreamPrep^®^ Freedom EVO^®^
Low-Throughput (<96)	Agilent Magnis
	Beckman Coulter Biomek NGeniuS
	PerkinElmer BioQule^™^ NGS System
	Tecan MagicPrep^™^

### 4.1. System compatibility

Ensuring a library preparation protocol is compatible with an automated platform can have a big impact on selection. Identifying a liquid handling system that already has the established vendor-approved workflow that matches the preparation kit that will be used can eliminate the time and energy needed to design a customized method. Illumina and New England Biolabs are two examples of companies that have partnered with leading automation manufacturers, including Agilent, Tecan, and PerkinElmer, to establish automated workflows for their library preparation kits. This opens the door for a single automated instrument to be used for several different sequencing applications. Another factor to consider if already performing NGS, is whether any internal changes were made to the manufacturer’s procedure or use of a lab developed protocol. Usually, the lab end user will not be an expert in scripting automation. So, for any modifications the lab will need to discuss with the manufacturer to see if adjustments can be incorporated into the automated workflow. However, if a lab can foresee the need to continually configure or develop new protocols, it may be worth the effort to invest time in training on scripting or choose an instrument designed to support this feature. It is also important to consider what consumables can be used. Proprietary hardware and tips may be a limiting factor if items are in high demand and become backordered. The ability to use more generic plates and tips offers some flexibility.

### 4.2. System capabilities

Although fully integrated “walk-away” automation seems ideal because it can free up scientists for other tasks, it may not be realistic for every lab. Fully automated systems require more space, more complex algorithms, and can be costly. Partial automation can be as simple as an automated pipetting station programmed to transfer and mix reagents. This will still require more work but should cut down on the hands-on time and reduce potential errors when compared to complete manual prep. However, if looking to eliminate almost all hands-on interaction, it is best to look for all-in-one liquid handlers. As mentioned previously, these systems may include multi-channel pipetting heads, plate grippers for moving hardware across the instrument deck, orbital shakers, and plate magnets for bead clean-up steps. Additional features such as on-deck thermal cyclers or storage towers for consumables may not be standard, thus increasing costs. Some systems also incorporate an on-deck or attached sequencer, further minimizing manual interactions. However, these extra items take up deck space and may decrease sample throughput.

### 4.3. System capacity

Robots designed for small batch volumes may be ideal for low-volume laboratories or those that prioritize faster turnaround times. Instruments equipped to prep 96–384 samples will likely be beneficial for facilities of higher volumes that need to sequence larger batches, depending on the system’s design. However, batching may result in an increase in turnaround times. Consumable use may also be a factor to consider. Automation requires a large amount of disposable hardware. And whether preparing 8 or 96 samples, some platforms may use the same amount of pipette tips, tubes, and plates. Therefore, it may be more economical to opt for sequencing in larger batches.

## 5. Designing regulatory-compliant validations

While NGS has become a more widely used practice, especially in the public health laboratory space, it may be useful to consider the assay design and implementation to meet regulatory compliance. Many regulatory programs (e.g., CLIA, CAP, etc.) have begun making more specific guidance (9, 10) and there are several useful resources available to strategize a successful approach (11).

The following components for validation of an automated liquid-handling instrument have been defined using a combination of best practices outlined by others, while tailoring to the instrument and DNA library preparation kit used (Table 2; Hamilton Microlab STAR and Illumina DNA Prep Kit products). It is worth noting that validations for laboratory developed tests (LDT) that are solely for surveillance purposes require a lower burden than those for diagnostic purposes to meet regulatory compliance. Consideration should be given as to how results will be used when on-boarding sequencing tests and platforms. Additional comprehensive examples using other systems can be found to help design, develop, and implement across diverse settings and different laboratory setups (12, 13).

**Table 2:** Example sample set and criteria for validation of WGS for major bacterial pathogens.

**Validation criteria**		
Accuracy	**Specimen list** *Acinetobacter baumannii* *Camplyobacter jejuni* *Enterobacter cloacae* *Escherichia coli* *Klebsiella pneumoniae* *Listeria monocytogenes* *Neisseria gonorrhoeae* *Salmonella enterica Heidelberg* *Salmonella enterica Typhimurium* *Salmonella enterica Arizoniae* (run control)	**Quality control metrics to meet criteria** Correct genus/species identification Coverage (20-40X depending on the genus) Identification of characteristic antimicrobial resistance (AMR) gene **Other important QC metrics** GC Content Number of Contigs 10 sample prepared and sequenced on a single run
Precision	Intra-Assay	5 samples prepared in duplicate and sequenced on single run and evaluated for QC metrics used in accuracy analysis
	Inter-Assay	5 samples prepared by 2 independent scientists, sequenced on separate MiSeq instruments, and evaluated for QC metrics used in accuracy analysis
	Method Comparison	5 samples prepared using manual and automated protocols, sequenced on single run, and evaluated for QC metrics used in accuracy analysis
Analytical sensitivity	DNA Input Range	1 ng - 10 ng*
	Limit of detection (LOD) determination	Run control subspecies ID with minimum accepted coverage (≥30X)
Analytical specificity	Secondary species abundance	≤ 1% of all sequencing reads are that of a “contaminant”

* Illumina recommends a standardized minimum DNA input (1 ng, extract concentration 0.2 ng/μL), which is required to obtain pipeline submittable sequencing read files.

### 5.1. Accuracy

Defined here as a measure of agreement between the tested sample and a reference, assessed for the following:

- Wet lab – sequencing platform (e.g., Illumina MiSeq, Oxford Nanopore, PacBio, etc.)
- Dry lab – bioinformatics pipelines

We validated the use of one platform and compared results from two pipelines to generate the accuracy of the assay. We prepared 10 samples and sequenced them on a single run to measure accuracy of this LDT.

### 5.2. Precision

Defined here as a measurement of consistency between the tested sample when run multiple times, under different conditions (e.g., days, operators, sample preparations, etc.). The number of samples required to meet this criterion should be at the direction and approval of each individual laboratory’s director. We utilized 5 samples to measure precision, as this was the minimum needed to test the range of organisms we test routinely, while also accounting for cost of supplies, reagents, and instrument use.

- Repeatability (Intra-assay precision) – samples tested in duplicate or triplicate within a single run
  **Note:** Be aware of potential sequencing biases or errors that can occur when there is too much similarity between samples.
- Reproducibility (Inter-assay precision) – samples prepared by individual operators on separate days, sequenced on the same run and/or on different runs

### 5.3. Sensitivity

Defined here as the limit of detection (LOD), we utilized the final concentration (High-sensitivity Qubit reading in ng/μL) of a prepared sample library that could be used to identify to species level.

### 5.4. Specificity

Defined here as the ability of a bioinformatics pipeline to identify contamination or interfering substances, as well as exclusion of a genus and/or species outside of those intended.

### 5.5. Method comparison (manual vs. automated protocols)

We added method comparison to determine if the results obtained from the new automated process differed from those using the currently validated manual preparation protocol. This was used solely to test the library preparation portion of the protocol, as the extraction method and the bioinformatics pipeline for analysis were identical between both methods.

### 5.6. Reference interval

Defined here as the normal value expected to correctly identify the genus and species of a given Gram-negative bacterial panel. However, this metric could be defined differently based on the desired target and intended use for result reporting. One example may be the presence or absence of a specific target gene.

### 5.7. Reportable range

Defined here as the output result that may be used for reporting, generally to include the genus and species identified, but may also contain serotype or other information. Depending on the use of a result, additional parameters with strict thresholds may be required, including coverage, Q30 scores, read length, etc.

## 6. Discussion

As we come out of the COVID-19 pandemic, it has become obvious that public health laboratories need to be ready to handle the next outbreak. The emergence of novel pathogens and the expansion of known antimicrobial resistant threats will likely balloon the test burden within public health over the coming decades. Sequencing, including automation, is just beginning to address public health needs and to aid in clinical diagnosis and treatment decisions. Working together with research, commercial, and clinical laboratories is essential to ensure a seamless transition from discovery and design to diagnosis, practice, and scaling. Advancement in NGS automation should be expected to continue, thus making new systems and instruments more prevalent, especially as they become more efficient and economical. Therefore, many public health laboratories should begin to consider the platforms and technologies that may work best for their workers, patients, and budgets.

As discussed previously, automation is essential to build testing capacity and to reduce the workload of manual test procedures. Although reliable and effective, automation can be complex and may bring new learning challenges to be of use. We recommend public health laboratories extend patience when acquiring new instrumentation, practice flexibility and generosity with time and resources that may be required for successful implementation and be communicative with others to problem-solve and troubleshoot. Automation is becoming more commonplace and there is an ever-growing network of laboratories and public health spaces that can work together to ensure the uptake and application of automation will continue to be valuable and successful.

## Data availability statement

The original contributions presented in the study are included in the article/supplementary material, further inquiries can be directed to the corresponding author.

## Author contributions

JS and VS conceptualized the topic and wrote equal sections of the manuscript. All authors contributed to manuscript revision, read, and approved the submitted version.

## Conflict of interest

The authors declare that the research was conducted in the absence of any commercial or financial relationships that could be construed as a potential conflict of interest.

## Publisher’s note

All claims expressed in this article are solely those of the authors and do not necessarily represent those of their affiliated organizations, or those of the publisher, the editors and the reviewers. Any product that may be evaluated in this article, or claim that may be made by its manufacturer, is not guaranteed or endorsed by the publisher.
